# The Effect of Number of Arms on the Aggregation Behavior of Thermoresponsive Poly(*N*‐isopropylacrylamide) Star Polymers

**DOI:** 10.1002/cphc.202000273

**Published:** 2020-06-02

**Authors:** Kaizheng Zhu, Ramón Pamies, Nodar Al‐Manasir, José Ginés Hernández Cifre, José García de la Torre, Bo Nyström, Anna‐Lena Kjøniksen

**Affiliations:** ^1^ Faculty of Engineering Østfold University College P.O. Box 700 1757 Halden Norway; ^2^ Department of Material Engineering and Manufacturing Technical University of Cartagena Cartagena Murcia 30202 Spain; ^3^ Mapei AS Sagstua 2120 Norway; ^4^ Department of Physical Chemistry University of Murcia Murcia 30100 Spain; ^5^ Department of Chemistry University of Oslo P.O. Box 1033, Blindern 0315 Oslo Norway

**Keywords:** dynamic light scattering, Monte Carlo simulations, poly(*N*-isopropylacrylamide), star polymers, thermoresponsive

## Abstract

The thermoresponsive nature of aqueous solutions of poly(*N*‐isopropylacrylamide) (PNIPAAM) star polymers containing 2, 3, 4, and 6 arms has been investigated by turbidity, dynamic light scattering, rheology, and rheo‐SALS. Simulations of the thermosensitive nature of the single star polymers have also been conducted. Some of the samples form aggregates even at temperatures significantly below the lower critical solution temperature (LCST) of PNIPAAM. Increasing concentration and number of arms promotes associations at low temperatures. When the temperature is raised, there is a competition between size increase due to enhanced aggregation and a size reduction caused by contraction. Monte Carlo simulations show that the single stars contract with increasing temperature, and that this contraction is more pronounced when the number of arms is increased. Some samples exhibit a minimum in the turbidity data after the initial increase at the cloud point. The combined rheology and rheo‐SALS data suggest that this is due to a fragmentation of the aggregates followed by re‐aggregation at even higher temperatures. Although the 6‐arm star polymer aggregates more than the other stars at low temperatures, the more compact structure renders it less prone to aggregation at temperatures above the cloud point.

## Graphical abstract

**How many arms**? Aqueous solutions of poly(*N*‐isopropylacrylamide) (PNIPAAM) star polymers exhibit a complex thermosensitive behavior. Increasing the number of arms promotes aggregation below the cloud point. When the temperature is raised, there is a competition between size increase due to enhanced aggregation and a size reduction caused by contraction. Some of the samples exhibits fragmentation of the aggregates at temperatures above the cloud point, followed by re‐aggregation at even higher temperatures.

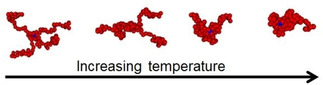

## 1 Introduction

Thermoresponsive polymers are interesting for numerous applications such as drug and gene delivery,^1^, ^2^, ^3^ imaging of cancer cells and tumors,^3^, ^4^, ^5^ tissue engineering,^6^ extraction of oil from sand,^7^ enhanced oil recovery,^8^ energy saving devices,^9^, ^10^ and desalination of seawater.^11^, ^12^ One of the most studied thermosensitive polymer is poly(*N*‐isopropylacrylamide) (PNIPAAM), which has a lower critical solution temperature of about 32 °C.^13^ The thermoresponsive nature of PNIPAAM has been explained by the formation of hydrogen bonds between the polymer and the surrounding water molecules.^14^ When the temperature is raised, the hydrogen bonds are broken, causing a coil‐to‐globule collapse of the polymer chains.^14^, ^15^ For low molecular weight PNIPAAM, the cloud point (CP) has been found to be dependent on both concentration and molecular weight.^16^ Co‐polymerizing with other polymers has a significant influence on the aggregation behavior of PNIPAAM.^17^, ^18^, ^19^ In addition, the terminal groups of PNIPAAM will affect its behavior.^20^, ^21^, ^22^ In view of this, it is interesting to examine whether the associative nature of this polymer is affected by the molecular architecture.

While the CP of branched PNIPAAM has been observed to decrease compared to the linear polymer,^23^ star polymers exhibit a more complex behavior. In some studies it was reported that the CP decreases with length of PNIPAAM star polymer arms.^24^, ^25^ However, the opposite tendency has also been observed.^26^, ^27^ The surprising rise in CP as the length of the arms (and the molecular weight) increases, has been explained by the presence of a hydrophobic core and by a high PNIPAAM chain density close to the core of the PNIPAAM stars.^26^, ^27^ Increasing the number of arms of PNIPAAM stars has been reported to lower the CP,^26^ due to a high local chain density close to the core of the PNIPAAM stars affecting their ability to form hydrogen bonds with water.^26^

Although studies of PNIPAAM star polymers have been conducted,^20^, ^22^, ^24^, ^25^, ^26^, ^27^ the effect of the number of arms on the temperature dependent self‐assembly of these polymers has not been conclusive. We previously reported a small angle X‐ray scattering (SAXS) study of PNIPAAM star polymers containing 2, 3, 4, and 6 arms^28^ (for the sake of coherence in the nomenclature the linear structure is denoted as a 2‐arm star). The SAXS experiments were conducted at relatively high polymer concentrations (1.0–5.0 wt %). In the present study, we are examining the same star polymers at low concentrations (0.01–1.0 wt %) utilizing dynamic light scattering (DLS), turbidity measurements, rheology, and rheo‐SALS (small angle light scattering under shear conditions). In addition, we have utilized Monte Carlo (MC) simulations to examine the effect of temperature on the conformation of the single star polymers. The combination of several experimental methods provides us with interesting information regarding the thermoresponsive nature of these star polymers.

## Experimental Section

### Materials and Sample Preparation

**Figure 1:**
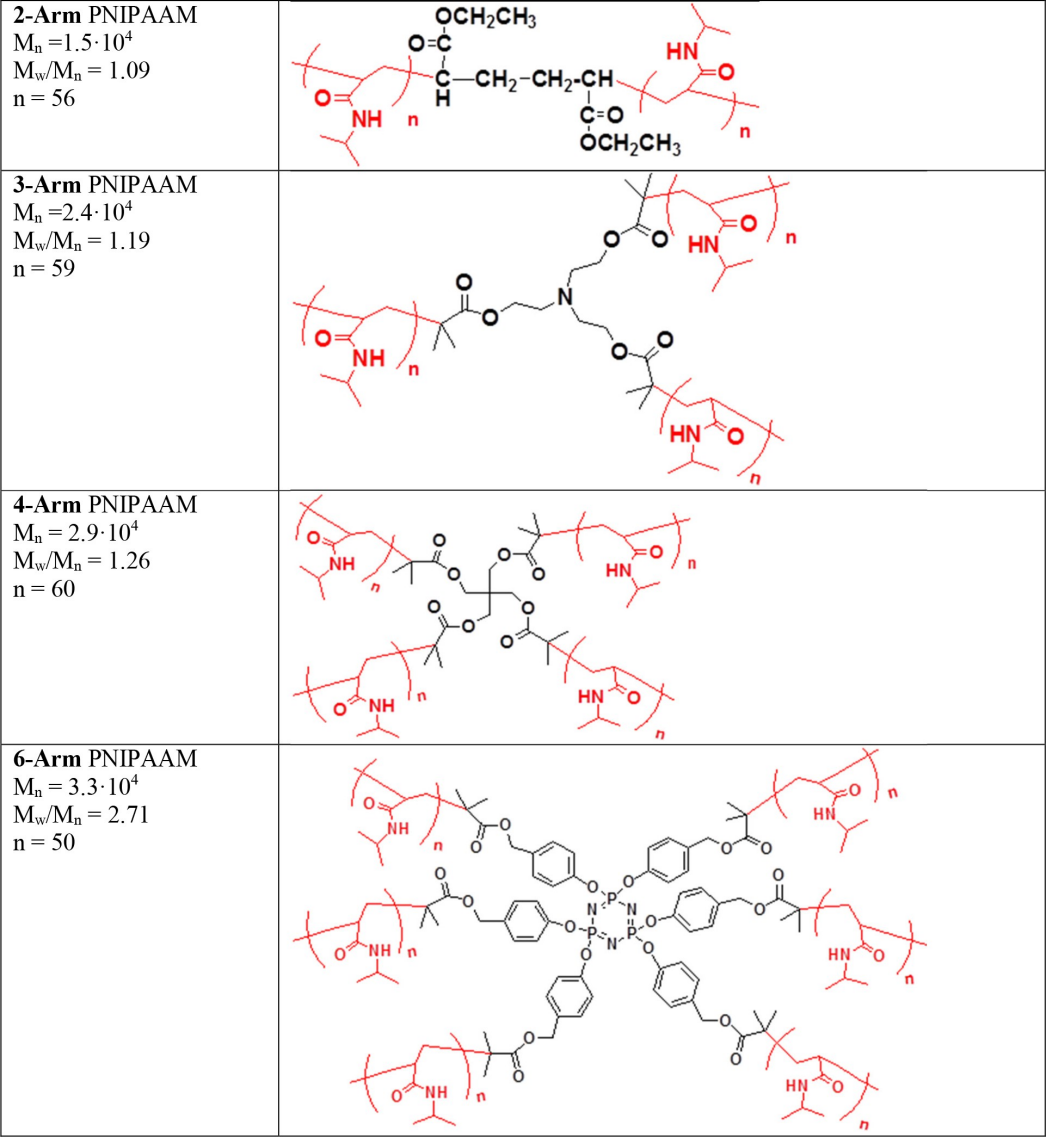
Number average molecular weights (M_n_), polydispersity index (M_w_/M_n_), length of the PNIPAAM arms (n), and structural formulas of the PNIPAAM star polymers are depicted.^28^

The procedure for the synthesis of the PNIPAAM star polymers has been reported previously.^28^ The differences in arm length and the relatively high polydispersities are due to the synthesis process, which render it difficult to control these parameters accurately enough to avoid any variation. In addition, the cores are different since they are adapted to the number of attached arms. Although these aspects can influence the associative behavior of the star polymers, the number of arms is believed to be the determining factor. The structure and molecular weight of the samples are shown in Figure 1. The ATRP synthesis procedure utilized here results in halide end groups. The samples were prepared at the desired concentration by weighting the components. The polymers were dissolved in purified Millipore Milli‐Q water, and stirred at room temperature until homogeneous solutions were obtained (for at least 24 h). All experiments were conducted with a heating rate of 0.2 °C/min.

### Turbidity

The turbidity was determined utilizing a NK60‐CPA cloud point analyzer (Phase Technology, Richmond, BC, Canada). The sample (0.15 mL) is placed by a micropipette on a glass plate coated by a high reflectivity metallic layer. The temperature of the sample cell is controlled by Peltier elements. To avoid solvent evaporation during the temperature scans, the sample is covered by the same amount of silicon oil. The light beam from a light source (AlGaAs, 654 nm) is focused on the sample by means of a lens. The scattered intensity signal (S) is detected by a light scattering detector located directly above the sample. The turbidity (τ) and the scattered intensity signal are related to each other by the empirical equation^29^ τ=9.0×10^−9^S^3.751^. The samples were measured with a heating rate of is 0.2 °C/min. The cloud points (CP) were determined as the temperature at which the first deviation of the scattered intensity from the baseline was observed.

### Dynamic Light Scattering

The dynamic light scattering (DLS) experiments were carried out by an ALV/CGS‐8F goniometer system, with 8 fiber‐optical detection units, from ALV‐GmbH, Langen, Germany. The polymer solutions were filtered in an atmosphere of filtered air through a 5 μm syringe filter (Millipore) directly into precleaned 10 mm NMR tubes (Wilmad Glass Co.).

Assuming that the scattering of the incoming light exhibit Gaussian statistics, the experimentally recorded intensity autocorrelation function g^2^(q,t) is directly linked to the theoretically amenable first‐order electric field autocorrelation function g^1^(q,t) through the Siegert relationship:^30^ g^2^(q,t)=1+B|g^1^(q,t)|^2^, where B (≤1) represents an instrumental parameter, and the magnitude of the wave vector, q, is q=(4πn/λ) sin(θ/2), λ is the wavelength of the incident light in a vacuum, θ is the scattering angle, and n is the refractive index of the medium.

Depending on the conditions, the correlation functions were found to exhibit either one or two relaxation modes. The correlation functions with two relaxation modes were fitted by the sum of a single and a stretched exponential: g^1^(q,t)=A_f_ exp(−t/τ_f_)+A_s_ exp[−(t/τ_se_)^β^] where A_f_+A_s_=1. The parameters A_f_ and A_s_ are the amplitudes and τ_f_ and τ_se_ are the relaxation times for the fast and the slow relaxation modes, respectively. The parameter τ_se_ is an effective relaxation time, and the stretched exponent β (0<β≤1) is a measure of the width of the distribution of the relaxation times. The mean slow relaxation time is given by τ_s_=(τ_se_/β)Γ(1/β), where Γ is the gamma function. The correlation functions where only one mode was evident were fitted by g^1^(q,t)=exp[−(t/τ_se_)^β^].

The apparent hydrodynamic radii have been calculated utilizing the Stokes‐Einstein relationship R_hf_=(k_B_T)/(6πη_0_D_f_); R_hs_=(k_B_T)/ (6πη_0_D_s_); where k_B_ is Boltzmann's constant, η_0_ is the viscosity of the solvent (water), T is the absolute temperature, and the mutual diffusion coefficients of the fast and slow mode is given by D_f_=1/(τ_f_q^2^) and D_s_=1/(τ_s_q^2^), respectively.

### Rheology and Rheo Small Angle Light Scattering (Rheo‐SALS)

Combined rheological and small angle light scattering experiments during shear flow were performed using the Paar‐Physica MCR 300 rheometer, equipped with a specially designed parallel plate‐plate configuration (the diameter of the plate is 43 mm) in glass. The instrumentation for the rheo‐SALS experiments was purchased from Physica‐Anton Paar. The sample was applied onto the lower plate by filtering the solution through a 5 μm Millipore filter. The distance between the plates is 0.5 mm. A 10 mW diode laser operating at a wavelength of 658 nm was used as the light source. The laser beam is passed through the sample placed between the transparent parallel plates. The forward scattered light at small angles was collected on a flat translucent screen below the sample.

The two‐dimensional scattering patterns formed on the screen were captured using a CCD camera (driver LuCam V. 3.8). A Lumenera (VGA) CCD camera (Lumenera Corporation, Ottawa, Canada) with a Pentax lens was utilized, and the scattered images were stored on a computer using the StreamPix (NorPix, Montreal, Quebec, Canada) application software (version 3.18.5), which enables a real‐time digitalization of the images. The images were acquired via the CCD camera with an exposure time of 200 ms. The pictures were analyzed utilizing a homemade software. The scattering patterns and viscosities were recorded continuously utilizing a heating rate of 0.2 °C/min at constant shear rates of 10 s^−1^ and 100 s^−1^. In addition, the scattering patterns were recorded in the absence of shear forces (0 s^−1^) utilizing the same heating rate.

### Simulations

In previous works,^17^, ^31^ MC simulations of linear PNIPAAM homo and heteropolymers have been performed. The calculations were conducted with the program MONTEHYDRO,^32^ which is freely available at http://leonardo.inf.um.es/macromol/ and implements the rigid‐body treatment to calculate hydrodynamic properties.^33^ With this approach, the polymeric chains are treated as having instantaneous rigid conformations to calculate their overall hydrodynamic properties. Thus, a set of conformations of the model chain is generated randomly following certain statistical rules (i. e. a MC procedure), and then the conformational properties of each conformation are evaluated using the procedures applicable to rigid particles and the final results are taken just as sample averages. The description of the friction of each element of the model and the computation of the hydrodynamic interaction between them is less complicated when these elements are spherical. Therefore, a simple and convenient way to build the polymeric chain is the usage of beads as elements. Springs with a suitable potential energy have been employed to connect these beads, which gives rise to the so‐called bead‐and‐spring model. The elements of the model must be parameterized to adequately represent star polymers. Two kinds of beads are required due to the presence of arms and cores with a different nature. For the sake of a better computational efficiency, an optimal number of two monomers per bead can be used without affecting the final result. In the works previously mentioned,^17^, ^31^ a multiscale approach was used to parameterized both the beads and springs from atomistic simulations performed with the commercial program HYPERCHEM distributed by Hypercube, Inc. (http://www.hyper.com/). There, the hydrodynamic radius of the two monomers forming a bead of the PNIPAAM chain, *σ*, was calculated after the atomistic simulations with the program HYDROPRO^34^ (freely available at http://leonardo.inf.um.es/macromol/) (see Table 1 in Schmidt et al.^31^). The same procedure is used here to assign a hydrodynamic radius to the bead that represents the star core. On the other hand, the part of the PNIPAAM chain that defines a spring (i. e. four monomers) was simulated at atomic level to obtain the distribution function of the spring length from which the spring parameters can be deduced (see Table 1  in Schmidt et al.^31^). Since the core bead is almost the same size as every arm bead and, furthermore, the number of arm‐core springs is negligible in comparison to the total number of springs, we considered, for the sake of simplicity, the same parameter values for all of the springs in the star. Finally, in order to address the temperature‐induced contraction of these thermosensitive polymers, the thermodynamical conditions (excluded volume) of the system are simulated using an intramolecular Lennard‐Jones potential as described previously,^31^ where typical values for the Lennard‐Jones parameters *σ*_LJ_ and *ϵ*_LJ_ can be found. The solvent quality (from good to poor) depends upon the ratio *ϵ*_LJ_/k_B_T, where k_B_T is the Boltzmann factor. Thus, varying that ratio is equivalent to varying the temperature of the thermosensitive polymer solutions.

**Table 1:** Simplified summary of the effect of concentration and the number of arms of the star polymers. ↑ indicates increasing values/tendencies, and ↓ indicates decreasing values/tendencies, ∩ indicates goes through a maximum.

	Increasing number of arms	Increasing concentration
Cloud point (CP)	↓	↓
Hydrodynamic radius of single stars	↑	–
Packing factor at low temperatures	↓	–
Packing factor at high temperatures	↑	–
Turbidity at low temperatures	↑	↑
Turbidity at high temperatures	↓	↑
Formation of aggregates below the CP	↑	↑
Size of aggregates above CP	∩	–

## 2 Results and Discussion

### 2.1 Turbidity

**Figure 2:**
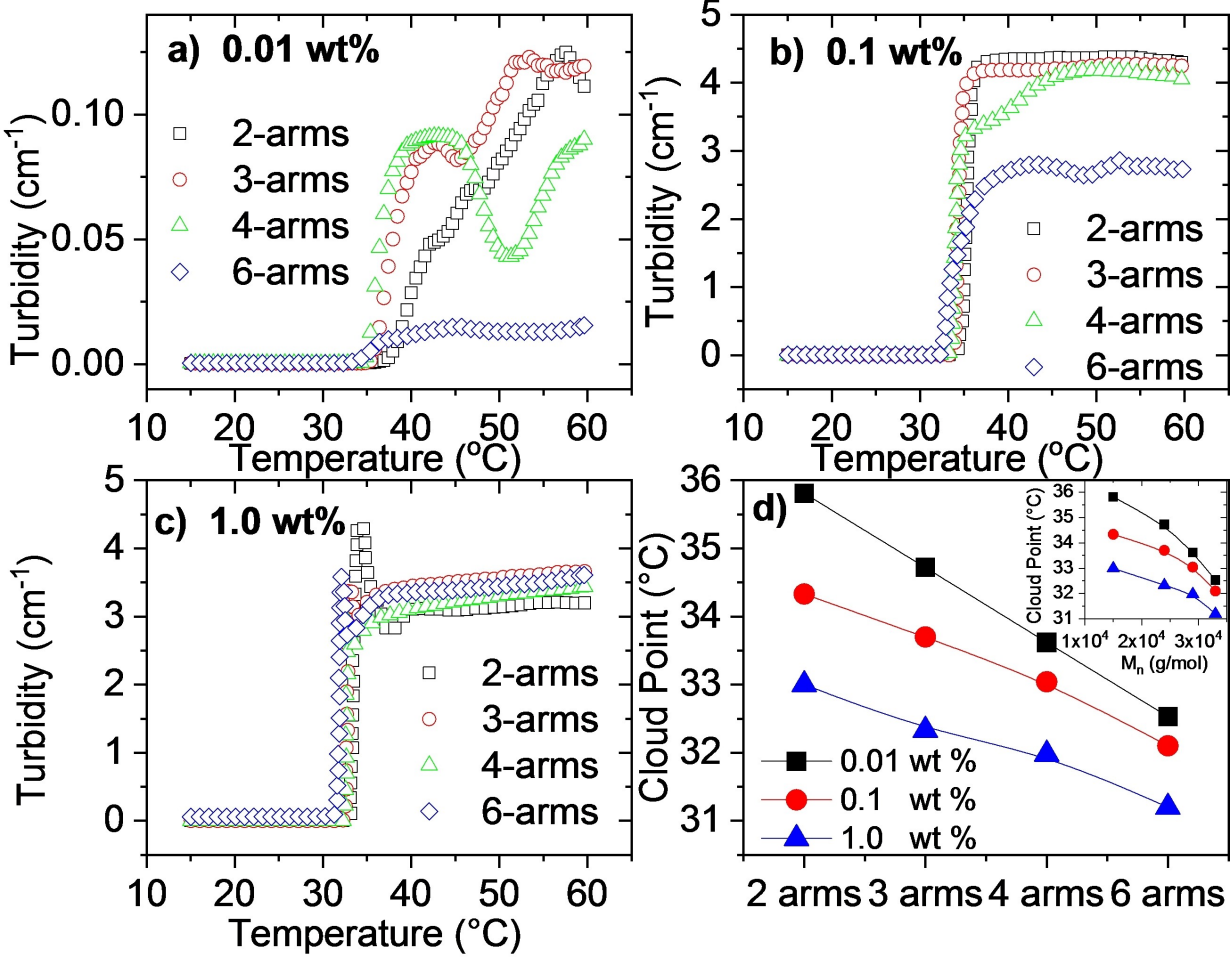
Turbidity as a function of temperature for the indicated systems measured at a heating rate of 0.2 °C/min for a) 0.01 wt %, b) 0.1 wt %, and c) 1.0 wt %. d) Cloud points for the indicated systems as a function of the number of arms; the inset plot illustrates how the cloud points vary as a function of the molecular weight. The lines are only guides for the eyes. The error bars are smaller than the size of the symbols.

The effect of the number of arms of the PNIPAAM star polymers were investigated at 3 different concentrations ranging from 0.01 to 1.0 wt %. Due to the thermoresponsive nature and lower critical solution temperature (LCST) it is interesting to first examine how the turbidity changes with temperature for these samples. Since the turbidity is directly proportional to the concentration, the samples at the lowest concentration never become visibly turbid. However, as can be seen from Figure 2a, there is still a measurable increase in turbidity at elevated temperatures. As illustrated by Figure 2, several of the samples exhibit a complex turbidity behavior where the turbidity first goes through a maximum, followed by a minimum in the turbidity values. Similar trends have been observed for several thermoresponsive polymers previously.^16^, ^35^, ^36^, ^37^, ^38^, ^39^, ^40^, ^41^, ^42^, ^43^, ^44^ In order to understand the cause of the complex turbidity data, we need to compare them with results from other experimental techniques and simulations. The turbidity data will therefore be discussed in more detail below.

Figure 2d shows the cloud points (CP) of the systems, determined as the temperature where the initial increase in turbidity is observed. As expected, the cloud point decreases with increasing concentration due to enhanced associations. When the number of arms and thereby the molecular weight of the PNIPAAM star polymer is raised, the cloud point is shifted towards lower values. It is however unclear whether this is due to the higher number of arms, or if it may be due to the corresponding increase in molecular weight as the number of arms increases. A similar decrease of the cloud point with increasing molecular weight has been observed for low molecular weight linear PNIPAAM^16^ as well as for star‐shaped PNIPAAM with increasing length of the arms.^24^, ^25^ However, as mentioned in the introduction, an increase in arm length can also have the opposite effect on the CP.^26^, ^27^ Xu and Liu reported a decrease in CP with higher number of arms,^26^ which was explained in terms of that the hydrogen bonds between PNIPAAM and water being affected by the high local chain density close to the core of the PNIPAAM stars.

### 2.2 Simulations

**Figure 3:**
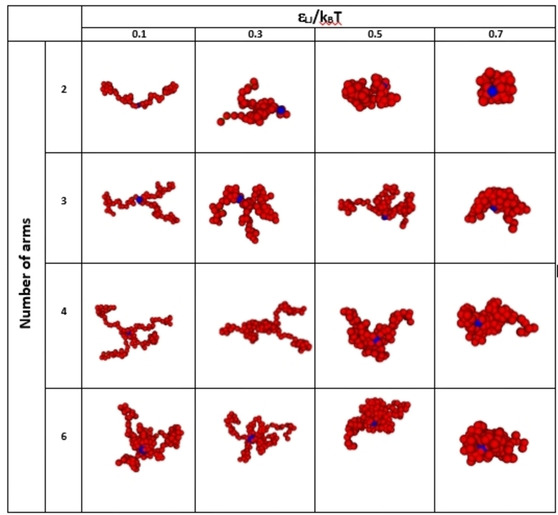
Pictures extracted from MC simulations for the PNIPAAM star polymers with increasing ϵ_LJ_/k_B_T value, i. e., representing increasing temperature.

In order to understand the aggregation behavior of the samples, it is instructive to examine how single, unassociated polymer star molecules behave as the temperature is changed. For that purpose, we have performed MC simulations of the four different star polymers at different solvent conditions (i. e. different *ϵ*_LJ_/k_B_T values), which in the simulations correspond to different temperature values. In the simulations *ϵ*_LJ_/k_B_T was varied from 0.1 (good solvent conditions) up to 2. However, results for values greater than 0.7 will not be presented since the structures were already fully collapsed and some bead overlap occurred that is not physically accepted for the simulation model. The core is considered to be at good solvent condition at any temperature. Therefore, *ϵ*_LJ_/k_B_T for the excluded volume interaction between the core and the arms is the geometrical mean of 0.1 and the *ϵ*_LJ_/k_B_T value ascribed to the arms interactions for a particular temperature.^31^ For every excluded volume interaction, the value *σ*_LJ_=0.51 nm is used according to the usual choice of setting *σ*_LJ_ to 0.8 times the equilibrium length of the connector springs.^31^ Figure 3 illustrates how the conformations of the star polymers change as the temperature is raised, i. e., as *ϵ*_LJ_/k_B_T increases in the MC simulations. The red beads represent the thermoresponsive part of the star polymer (the PNIPAAM arms) whereas the blue beads correspond to the core. At good solvent conditions, i. e., *ϵ*_LJ_/k_B_T=0.1 (low temperatures), the chain conformation is quite open. At *ϵ*_LJ_/k_B_T>0.3 (increased temperatures), poor solvent conditions emerge, and a contraction of the star polymer occurs. When *ϵ*_LJ_/k_B_T=0.7 (high temperatures) the poor solvent conditions give rise to a chain collapse and a compact conformation (Figure 3).

**Figure 4:**
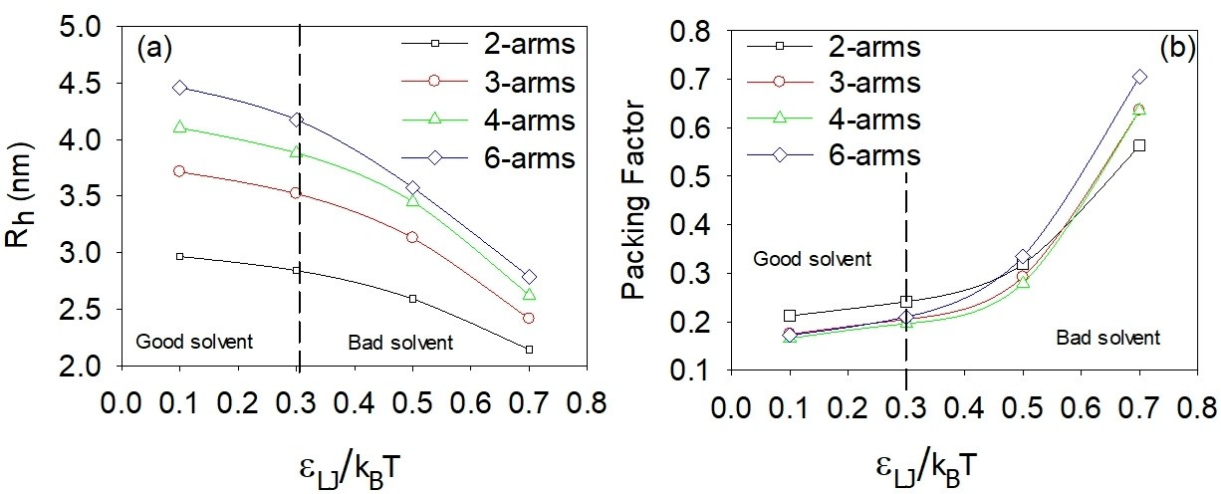
The hydrodynamic radius (a) and packing factor (b) of unassociated PNIPAAM star polymers as a function of solvent conditions, calculated from MC simulations. The lines are only guides for the eyes.

**Figure 5:**
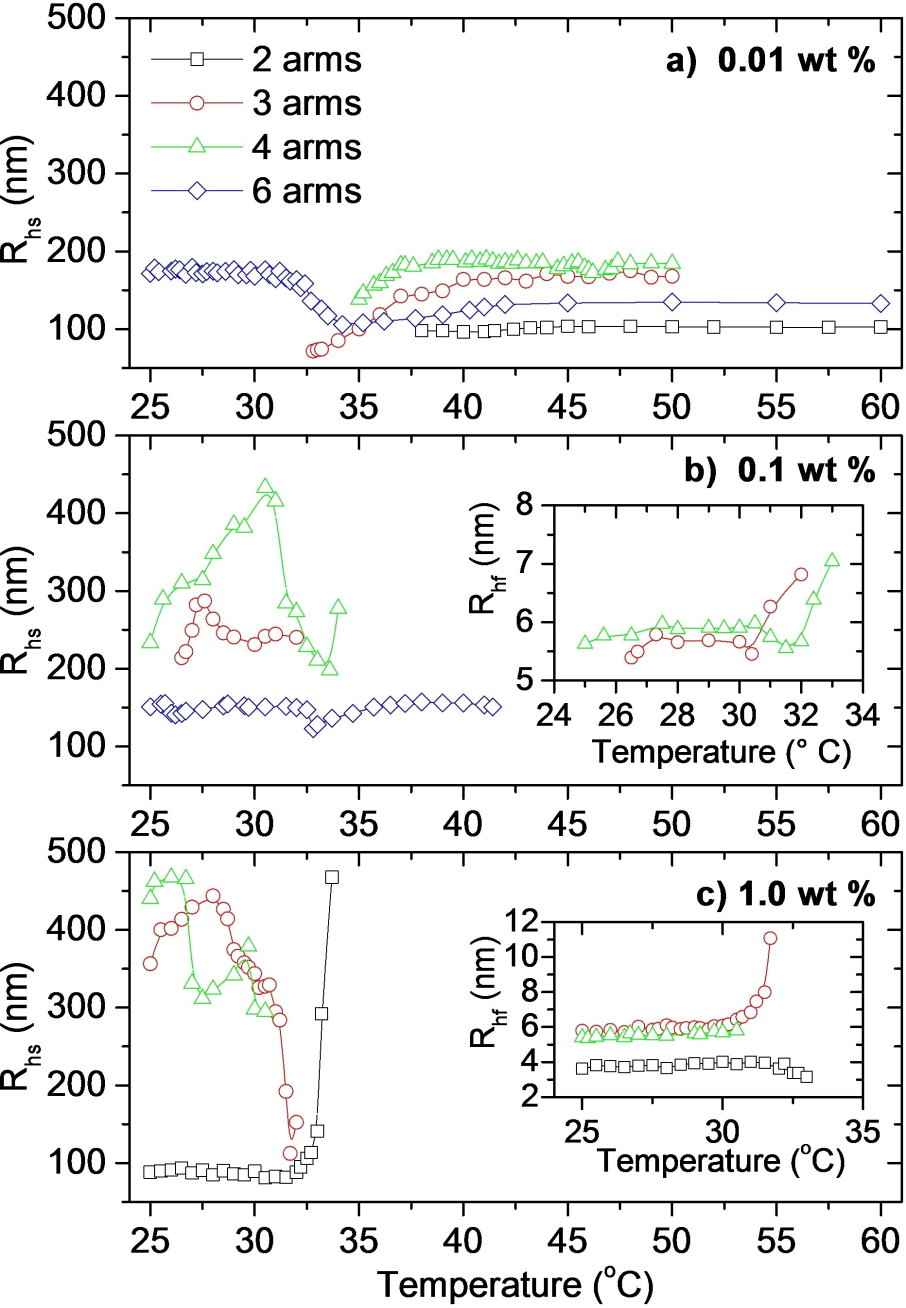
The apparent hydrodynamic radius determined by DLS from the slow relaxation mode (aggregates) for the indicated systems measured at a heating rate of 0.2 °C/min for a) 0.01 wt %, b) 0.1 wt %, and c) 1.0 wt %. The inset plots display the sizes determined from the fast relaxation mode.

Figure 4a shows the dependence of the hydrodynamic radius, R_h_, on the solvent condition (*ϵ*_LJ_/k_B_T) for the different unassociated star polymers as obtained from MC simulations. As expected, R_h_ decreases as the solvent conditions deteriorate, i. e., when the temperature is raised. At good solvent conditions, the values of R_h_ range from 3.0 nm (2‐arms star) to 4.5 nm (6‐arm star), these values agree quite well with the experimental data from DLS presented below (see insets in Figure 5). Interestingly, the star polymers with a higher number of arms experience the maximum relative contraction. Thus, R_h_ of the 6‐arm star at good solvent conditions is 4.5 nm, whereas at very poor solvent conditions it is about 3.0 nm, close to the value found for the (originally smaller) 4‐arm star at the same poor solvent conditions. To evaluate the relative contraction of these polymers, we have defined a Packing Factor (PF) as follows:

PF=(Volume occupied by the beads)/(Hydrodynamic volume of the star), where the volume occupied by the beads is simply computed as the combined volumes of the individual beads forming the star, and the hydrodynamic volume is the volume of the sphere with the same hydrodynamic radius as the star. Figure 4b shows the variation of PF for the different stars as a function of the solvent condition. Interestingly, at good solvent conditions, 3‐arm, 4‐arm, and 6‐arm star polymers have similar PF values, which indicate that about 18 % of the hydrodynamic volume is occupied by the beads (i. e. by polymer mass). However, the 2‐arms star polymer exhibits a slightly more compact structure with about 20 % of the hydrodynamic volume occupied by the beads. As the temperature is raised (or *ϵ*_LJ_/k_B_T increased) the contraction of the 3‐arm and 4‐arm stars is similar, whereas the 6‐arm star experiences a steeper contraction (more pronounced increase of PF) and the 2‐arm star displays a weaker contraction. This different behavior gives rise to a crossover between the 2‐arm star (a linear chain) and the curves corresponding to the stars with a higher number of arms. As a consequence, at the highest considered *ϵ*_LJ_/k_B_T value, the most open structure is the 2‐arm star, whereas the 6‐arm star exhibits the most compact structure: 55 % of the hydrodynamic volume is occupied by polymer mass (beads) for the former and 70 % for the latter.

### 2.3 Dynamic Light Scattering

The apparent hydrodynamic radii determined from dynamic light scattering are displayed in Figure 5. As is evident from the figure, there are missing data from some of the systems at high and/or low temperatures, and a couple of curves are missing altogether. The missing data at low temperatures is mainly due to very low scattering intensities. The data is collected during a heating ramp. Accordingly, the accumulation time of each correlation function should be limited to relatively short times to avoid significant temperature changes during the data collection. For very low concentrations of low molecular weight samples that are not forming large aggregates, the scattered intensities are not high enough to provide data of a sufficient quality for analyses. At high temperatures, multiple scattering prevents analysis of the data for some of the samples. At the 0.1 wt % concentration, data from the 2‐arm polymer is missing altogether. This is caused by too low scattering intensities at low temperatures, multiple scattering at high temperatures, and a very sharp transition zone between these two regions (see Figure 2b). We were not able to analyze the data from the 6‐arm polymer at the highest concentration, due to multiple scattering throughout the whole temperature region.

At some conditions, the correlation functions obtained from dynamic light scattering exhibit two relaxation modes, indicating a bi‐modal size distribution. The apparent hydrodynamic radii calculated from the slow relaxation mode ranges from about 70 nm and up to nearly 500 nm, demonstrating the formation of large structures in the samples. The inset plots in Figure 5 display the sizes determined from the fast relaxation times. At the lowest temperatures, the hydrodynamic radii (R_hf_) determined from the fast relaxation mode is just a few nm, indicating unassociated polymer unimers in the solution.^16^, ^43^, ^44^, ^45^ These R_h,f_ values are in good agreement with the MC simulations results (Figure 4a). As can be seen from the inset in Figure 5c, the size of the 2‐arm star is slightly smaller than the 3‐ and 4‐arm stars. This is caused by the linear nature of the 2‐arm polymer facilitating a coil‐like conformation, combined with the somewhat lower molecular weight, as demonstrated by the simulation results. R_hf_ remains practically constant when the samples are heated, until the cloud point is approached. At this stage, the 2‐arm star (inset in Figure 5c) exhibit a size decrease due to a contraction of the polymer coils as the associative forces between the polymer chains becomes stronger.^16^, ^46^ The same effect is observed by the MC simulations (Figure 4a), and is in agreement with SANS experiments conducted by Hammouda et al.^47^ The enhanced associations also promote intermolecular associations, causing the previously unassociated polymer stars to stick to each other; raising the values of R_hf_ for the 3‐ and 4‐arm stars. The fast relaxation mode is not detected at the lowest polymer concentration. This is due to a very small concentration of low molecular weight polymers, causing too weak scattering intensities for detection. For the 6‐arm star, the absence of a fast mode at 0.1 wt % is caused by the strong associations in the sample, with large clusters dominating the scattering profile.

It is interesting to note that the star polymers form aggregates even at temperatures significantly below the CP of the systems. The low turbidity values at these conditions (Figure 2) and the conformational study (Figures 3 and 4), show that the aggregates have an open structure, i. e., they contain a high proportion of solvent. Accordingly, the difference in refractive index between the aggregates and the solvent is small and therefore causing low turbidity values.^44^, ^48^, ^49^ Formation of aggregates below the CP illustrates that the associative nature of these polymers are established even at low temperatures. Similar behavior has been observed for thermoresponsive polymers previously.^22^, ^42^, ^43^, ^44^, ^45^, ^47^, ^50^, ^51^, ^52^ When the attractive forces between the polymer chains are moderate, the intermolecular interactions are strong enough to cause aggregate formation, but the intramolecular interactions are not sufficient to induce a collapse of the aggregates into a compact structure. The aggregates formed at these conditions are therefore not compact enough to significantly raise the turbidity values (which will increase with the aggregate size and compactness).^44^, ^49^ Accordingly, even though aggregates are formed, no CP is evident at these conditions. As the temperature is raised further, the associative interaction becomes stronger, and the aggregates start to collapse resulting in higher turbidity values and thereby a CP. The co‐existence of the aggregates with a fast mode illustrates that only a fraction of the polymers is in the form of aggregates. This is in agreement with theoretical considerations of block copolymer associations,^53^ and with SANS measurements on deuterated/non‐deuterated PNIPAAM.^47^

It is reasonable to assume that the correlation functions that cannot be analyzed due to too low scattered intensities indicate the absence of aggregates (which scatter much more than the unassociated polymer chains). Accordingly, at the lowest polymer concentration only the 6‐arm polymer form aggregates below the CP. However, as the concentration is raised to 0.1 wt %, only the linear 2‐arm star is not aggregating below the CP, and at the highest concentration, associative behavior is observed at low temperatures for all of the considered systems.

**Figure 6:**
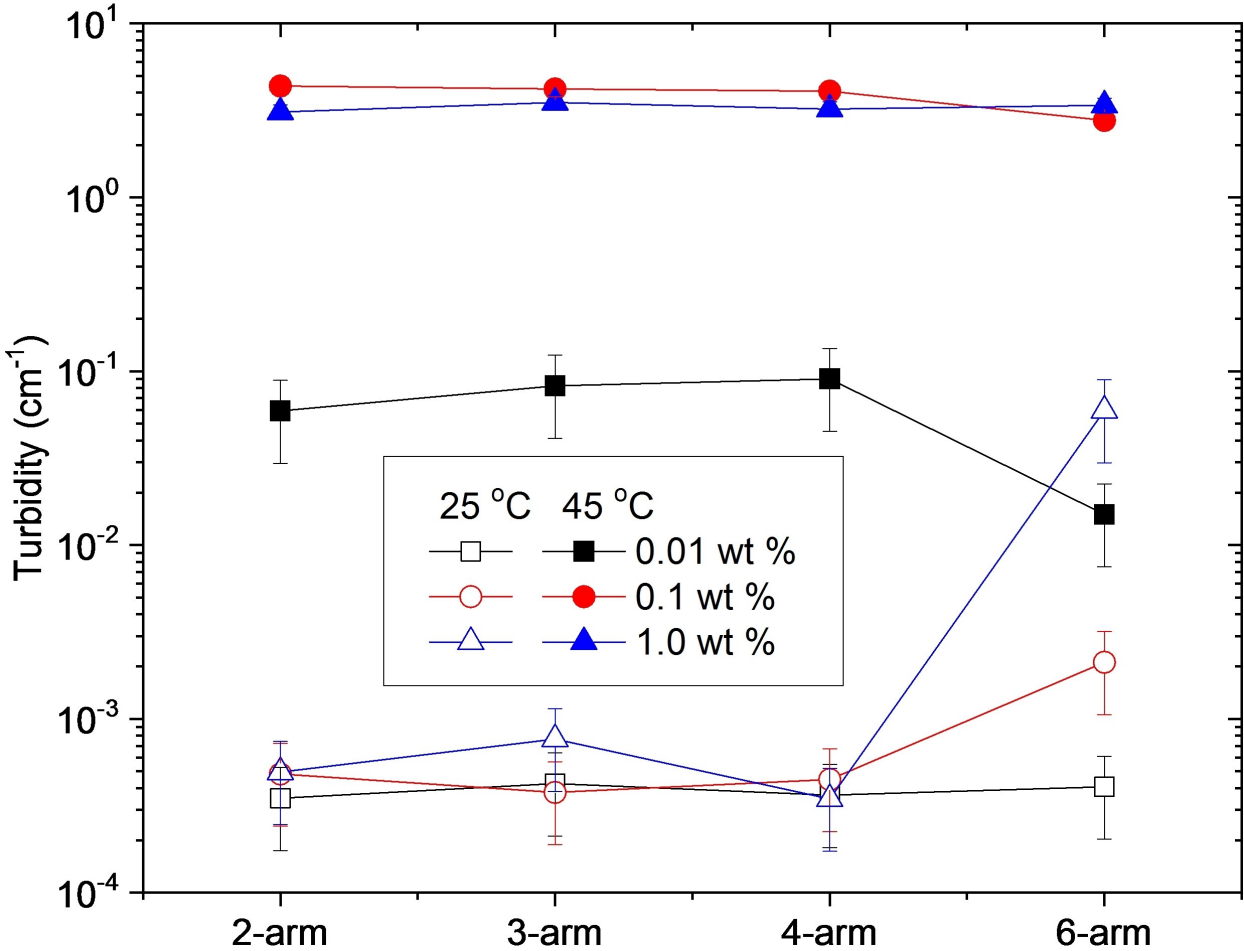
Turbidity values below the cloud points (25 °C), and above the cloud points (45 °C) for the indicted systems.

Formation of aggregates at temperatures significantly below the CP even at 0.01 wt % and multiple scattering even at low temperatures at 1.0 wt % show that the 6‐arm polymer has a stronger tendency to form aggregates at low temperatures than the polymers with fewer arms. This corroborates with the higher turbidity values at low temperatures for 0.1 and 1.0 wt % of the 6‐arm star polymer compared to the star polymers with a smaller number of arms (Figure 6). This raises the question of whether the enhanced associations below the CP are due to the somewhat higher molecular weight of the 6‐arm stars, or a result of the molecular architecture. Considering that at low temperatures the PF of the 6‐arm stars are very similar to the 3‐ and 4‐arm stars (Figure 4b), the difference is most likely due to the higher molecular weight of the 6‐arm stars.^16^ In the previous SAXS study^28^ on the same polymers, it was reported that over a broad temperature interval the values of the second virial coefficient A_2_ are lower for the 6‐arm star polymer and it was also found that A_2_ decreases with increasing molecular weight.

As can be seen from Figure 5, R_hs_ exhibit a complex temperature dependency. This is caused by the competition between intramolecular associations that cause contraction of the moieties and thereby size decrease, and intermolecular associations generating enhanced interchain aggregation, which will lead to a size increase.

Comparing the sizes obtained from DLS with the turbidity data in Figure 2, we can gain more information about the system than the techniques provide separately. We notice that at the lowest concentration, the aggregates formed by the 2‐arm star increase slightly in size just after the CP, before it levels off at higher temperatures (Figure 5a). However, even though the sizes remain constant above 45 °C, the turbidity continues to rise (Figure 2a). A changing turbidity value clearly demonstrates that something is occurring in the sample, even though the size remains constant. Both aggregation and contraction of the aggregates increase the turbidity values.^44^, ^48^, ^49^ These processes are both promoted by the enhanced associative nature of PNIPAAM at elevated temperatures. In this case, contraction combined with aggregation causes the sizes to remain constant as the two effects cancel each other out.^54^

At 0.01 wt %, the 3‐ and 4‐arm stars exhibit sizes that are clearly increasing at temperatures above the CP (Figure 5a), combined with the upturn in the turbidity values this is an indication of the growth of large aggregates. As for the 2‐arm star, the sizes level off at high temperatures. However, the turbidity data for the 3‐ and 4‐arm stars exhibit a more complex behavior. After the initial increase, there is a decrease in the turbidity before the values rise again at even higher temperatures. This is especially evident for the 4‐arm star, but a slight minimum is also observed for the 3‐arm polymer. This intriguing variation in turbidity is also observed at 1.0 wt %, and will be discussed in more details below.

As mentioned previously, the 6‐arm star polymer forms aggregates below the CP even at the lowest concentration. In the vicinity of the CP, the sizes decrease before becoming larger again (Figure 5a), while the turbidity increases (Figure 2a). This indicates a contraction of the initial aggregates followed by an aggregation‐induced size increase. For this polymer, both the size and the turbidity become nearly constant above 45 °C. Accordingly, unlike the other polymers, the aggregates formed by the 6‐star polymer at 0.01 wt % are stable at high temperatures. Comparing the sizes of the polymers at elevated temperatures for the lowest concentration (Figure 5a), it is evident that as the number of arms is raised from 2 to 4, the sizes of the aggregates increase. Before the turbidity values decline again, the turbidity increases as the number of arms increases to 4 (Figure 2a and Figure 6). This illustrates that a higher number of arms (up to 4) promotes aggregation above the CP. This is probably due to the increased molecular weight as the number of arms is raised.^16^

Interestingly, even though the 6‐arm star polymer has the highest molecular weight and the strongest associations at low temperatures, the polymer exhibits the lowest turbidities at high temperatures for the 0.01 and 0.1 wt % concentrations (Figure 2a,b and Figure 6). At a constant polymer concentration, the turbidity will increase when the aggregation number rises and when the particles become more compact.^44^, ^49^ Accordingly, compared to the other polymers the 6‐arm star polymer forms aggregates with a lower aggregation number and/or aggregates with more open spaces and/or fewer aggregates. According to the MC simulations, the 6‐arm stars have the highest packing factor at elevated temperatures (Figure 4b). It has been argued that the sticking probability is reduced for very compact structures.^44^, ^49^, ^55^, ^56^, ^57^ In view of this, it is reasonable to assume that at high temperatures the 6‐arm star polymer has reduced sticking probability. This would result in fewer aggregates and lower aggregation numbers, in agreement with the turbidity data. A reduced sticking probability can also explain why the 6‐arm stars form smaller aggregates than the 3‐ and 4‐arm stars at elevated temperatures (Figure 5a). However, the 2‐arm star polymer exhibits even lower sizes at these conditions. This is due to the stronger associative nature of the 6‐arm stars at low temperatures, which give rise to aggregate formation even before the sample is heated up. As the temperature is raised, the additional aggregation of the 6‐arm stars is modest, whereas the 2‐arm star polymer shows enhanced associations. While the sizes of the aggregates formed by the 6‐arm star polymer remain larger than those of the 2‐arm star polymer, the number of aggregates in the 2‐arm star polymer is higher, resulting in larger turbidity values.

When the polymer concentration is raised to 0.1 wt %, the turbidity transition is more abrupt (Figure 2b). This is due to enhanced associations at higher concentrations combined with a higher collision frequency when the number of entities in the solution increases. At this concentration, the 3‐, 4‐, and 6‐arm star polymers form aggregates even below CP (Figure 5b); for the 3‐ and 4‐arm polymers there is a size increase due to enhanced aggregation, followed by a decline in size that indicates contraction of the aggregates. At high temperatures, multiple scattering prevents data analysis for the 2‐, 3‐, and 4‐arm star polymers due to the formation of large, compact aggregates. The 6‐arm star polymer is much less turbid than the other polymers at high temperatures (Figure 2b), similar to what was observed at 0.01 wt %. As mentioned above, this is due to a reduced sticking probability of the 6‐arm star polymer. Accordingly, the 6‐arm polymer can be heated up to higher temperatures before multiple scattering occurs.

As mentioned previously, raising the concentration to 1.0 wt % causes multiple scattering from the 6‐arm star throughout the whole temperature region. At this concentration, the 2‐arm polymer forms relatively small aggregates at low temperatures (Figure 5c). The clusters increase in size around the CP, where the turbidity also becomes higher (Figure 2c); this demonstrates enhanced association of the system at these conditions. The 3‐ and 4‐arm star polymers exhibit a more complex size dependency at low temperatures, where large aggregates are formed (Figure 5c). The variation in size is due to a competition between enhanced aggregation and contraction of the aggregates.

At this concentration (1.0 wt %), all samples exhibit an initial increase in the turbidity, followed by decreased turbidity before the samples become more turbid again at higher temperatures (Figure 2c). Unfortunately, multiple scattering prevents analyses of the DLS data in this region. This intriguing turbidity trend has been observed for several thermoresponsive polymer systems before.^16^, ^35^, ^36^, ^37^, ^38^, ^39^, ^40^, ^41^, ^42^, ^43^, ^44^ Decreasing turbidity values can be caused by several effects. For very large and compact aggregates, Mie theory predicts that the turbidity will start to oscillate as the size of the aggregates grow larger. However, after the first minimum, the next maximum turbidity value should be lower than the first turbidity maximum.^48^, ^49^ Except for the 2‐arm polymer, the turbidity values are increasing back to the initial values. Unless some additional mechanisms come into play, this explanation does not fit the measured data for the other polymers. Swelling of the polymer aggregates would decrease the difference in refractive index between the sample and the solvent, and thereby lower the turbidities. However, since PNIPAAM has a stronger tendency to aggregate when the temperature is increased, contraction of the aggregates is much more likely than swelling, which corroborates with the packing factors obtained from the MC simulations (Figure 4b). In addition, the analysis of the SAXS data in the previous study^28^ of these polymers showed that a model of Gaussian star molecules, which neglects self‐avoidance within the molecules, is sufficient to describe the data for these samples at the considered conditions. Accordingly, the polymer blocks are not long enough to display excluded volume effects. Another effect that would lead to a decline of the turbidity is fragmentation of the clusters into smaller entities. Even though this might seem counterintuitive for associative systems, this has actually been observed for other thermoresponsive polymers previously.^36^, ^40^, ^41^, ^42^, ^43^, ^44^ However, in these earlier studies the fragmentation has been attributed to the formation of micellar‐like structures of block copolymers. This explanation does not seem reasonable for these PNIPAAM stars, but fractionation could still be a possibility. Additional measurements are needed in order to explore whether the turbidity decrease is due to fractionation. These effects will therefore be discussed in more detail in connection with the rheology and rheo‐SALS measurements below.

### 2.4 Rheology

**Figure 7:**
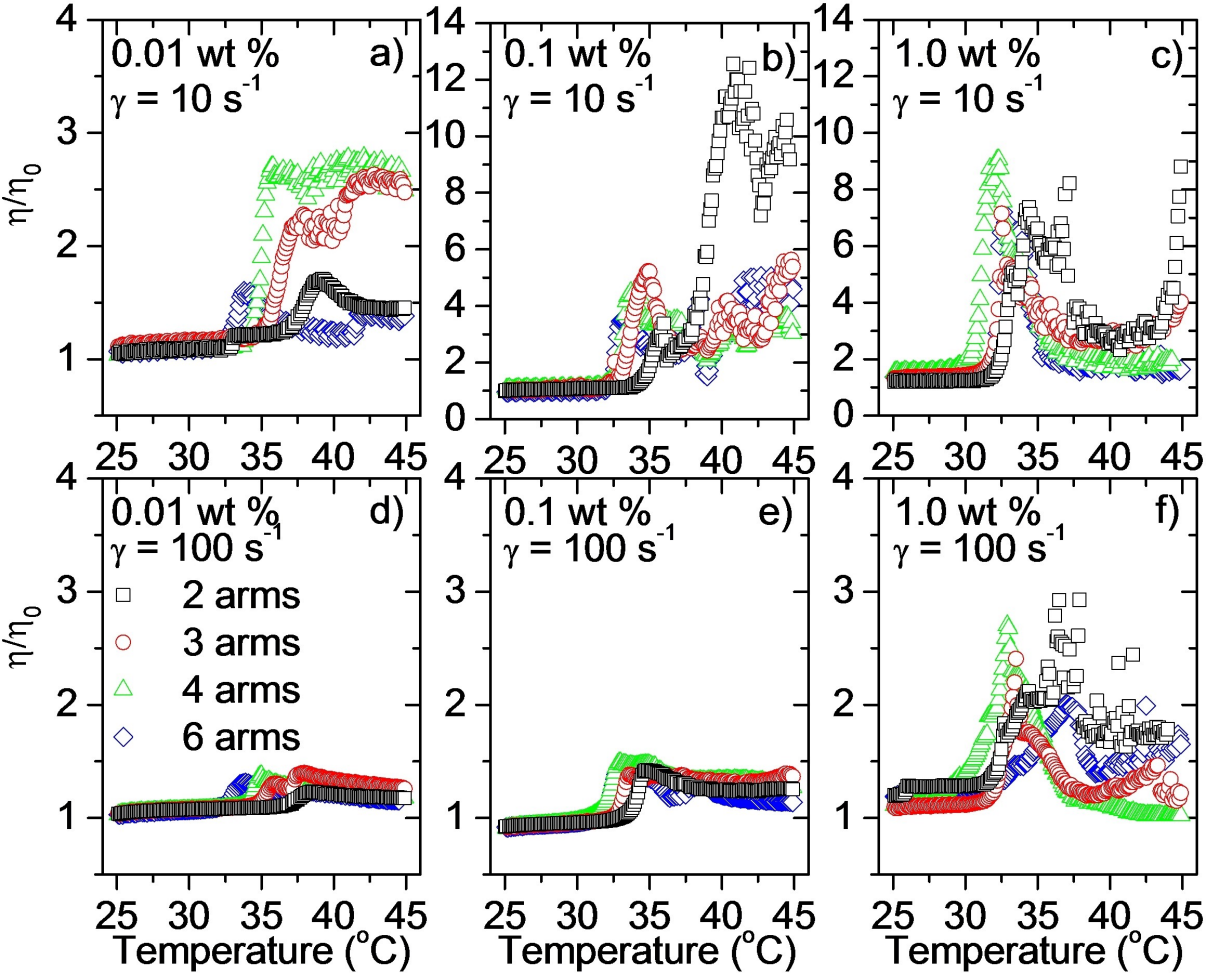
The reduced viscosity (viscosity of the sample divided by the viscosity of water) for the indicated systems. Measured at a heating rate of 0.2 °C/min at a constant shear rate of 10 s^−1^ (a,b,c) or 100 s^−1^ (d,e,f).

The reduced viscosities (η/η_0_) of the samples are displayed in Figure 7. The viscosities have been divided by the viscosity of water in order to separate the effect of the polymer samples from the declining viscosity of water with increasing temperature. The measurements are conducted at the same heating rate as for the turbidity and DLS experiments (0.2 °C/min) under the influence of a constant shear rate (10 s^−1^ and 100 s^−1^). An increase in the reduced viscosity indicates a build‐up of larger clusters in the samples.^58^ As can be seen from Figure 7 a,b,c, large aggregates are formed at elevated temperatures at a shear rate of 10 s^−1^. Comparing with Figure 7 d,e,f it is evident that these aggregates are broken down by the higher shear rate of 100 s^−1^ (note the different scale on the y‐axis of Figure 7 b,c compared with the other figures). A break‐down of the clusters at a moderate shear rate illustrates that they are not sufficiently strong to withstand the augmented shear forces applied to the samples. The aggregates are somewhat stronger at the highest concentration (Figure 7f), where there is a build‐up of large clusters even at the shear rate of 100 s^−1^.

For the highest concentration exposed to the lowest shear rate (Figure 7c), there is a clear maximum in the reduced viscosity values followed by a minimum before the reduced viscosities start to rise again. Accordingly, there is a build‐up of clusters, followed by a break‐down of the clusters before they are growing again. The profiles are reminiscent of the profile of the turbidity for the same samples (Figure 2c). This is in agreement with the conjecture that the complex turbidity behavior is a result of initial aggregation, followed by a fragmentation before the aggregates start to grow again. However, since the rheology measurements are conducted under shear conditions, which can influence the mechanism of aggregate formation, the rheological data and the turbidity values may not be completely comparable.

### 2.5 Rheo‐SALS

**Figure 8:**
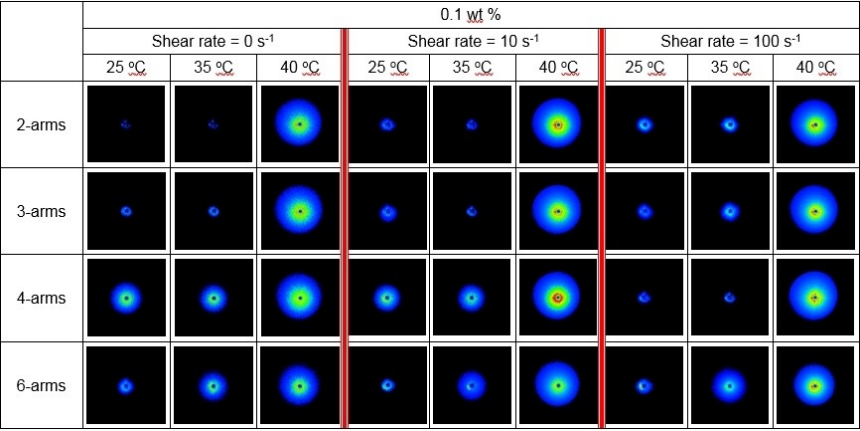
Rheo‐SALS scattering patterns for the indicated systems at a polymer concentration of 0.1 wt %. Conducted at a heating rate of 0.2 °C/min. The scattered intensities are converted to a color code where increasing scattered intensities go from blue at low scattered intensities through green and yellow towards red at high scattered intensities. The black spot in the middle of the pictures is the beam stop.

Rheo‐SALS experiments (small angle light scattering under the influence of shear forces) were conducted at the same time as the rheological experiments. Rheology measurements for these low‐viscosity samples are limited to relatively high shear rates due to experimental limitations. However, the rheo‐SALS were also conducted at zero‐shear conditions. The 2D scattering images for the 0.1 wt % samples at different shear rates are displayed in Figure 8 for a low, medium and high temperature. As expected, the scattered intensities increase as the temperature is raised.

**Figure 9:**
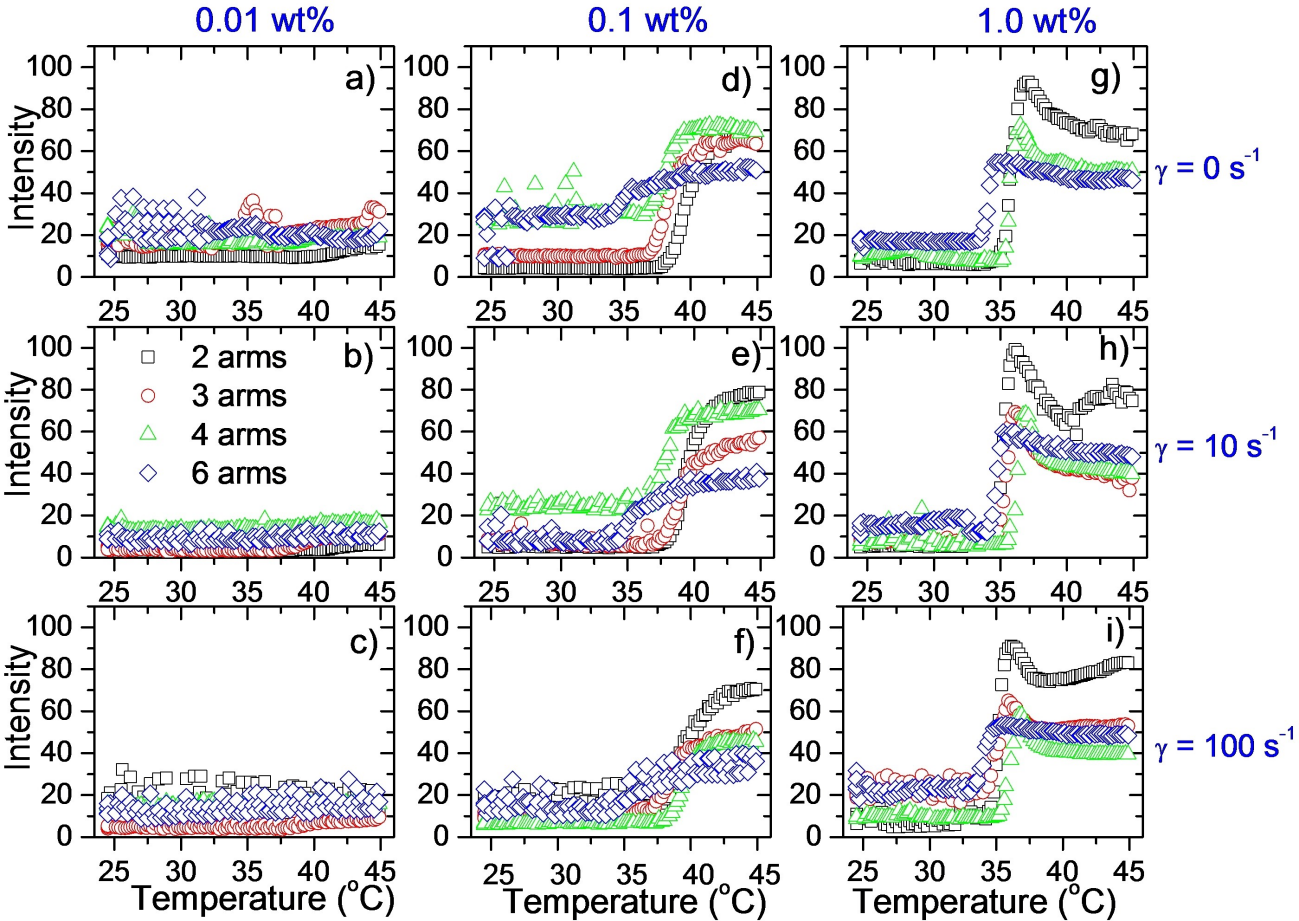
Rheo‐SALS scattered intensities (arbitrary units) integrated over the whole scattering pattern for the indicated systems. Conducted at a heating rate of 0.2 °C/min.

The total scattered intensities (integrated over the whole image) are shown in Figure 9. For the lowest concentration (Figure 9 a,b,c) the scattered intensities are too low for gaining any usable information. At 0.1 wt %, the intensities are increasing around the CP of the samples (Figure 9 d,e,f). For the highest concentration (Figure 9 g,h,i) the scattered intensities increase around the CP of the samples, followed by a decline at higher temperatures. The scattered intensities are dependent on several factors such as the polymer concentration, the size and shape of the aggregates, and the compactness/swelling of the aggregates (changes in refractive index of the aggregates). As can be seen from Figure 8, all the scattering patterns are circular (the same is the case for the other concentrations). This indicates that the large aggregates probed by this method are isotropic, i. e., approximately spherical. If elongated clusters were forming, these are expected to align in the shear direction thereby giving rise to an anisotropic (non‐circular) scattering pattern.^59^, ^60^, ^61^ Hence, the changes in scattered intensities are most likely due to changes in size and/or compactness of the aggregates. Comparing with Figure 7c, the scattered intensities in Figure 9h (same concentration and shear rate) are somewhat similar to the trends in viscosity values. Thus, the scattered intensities increase when the clusters are growing and decline when the clusters become smaller. This confirms that the decrease in viscosities is due to a fragmentation of the clusters.^44^, ^49^ Since a corresponding maximum in scattered intensity is also observed in the absence of shear forces (Figure 9g), the observed turbidity maximums (Figure 2c) is caused by fragmentation of the clusters, before they re‐aggregate at even higher temperatures.

**Figure 10:**
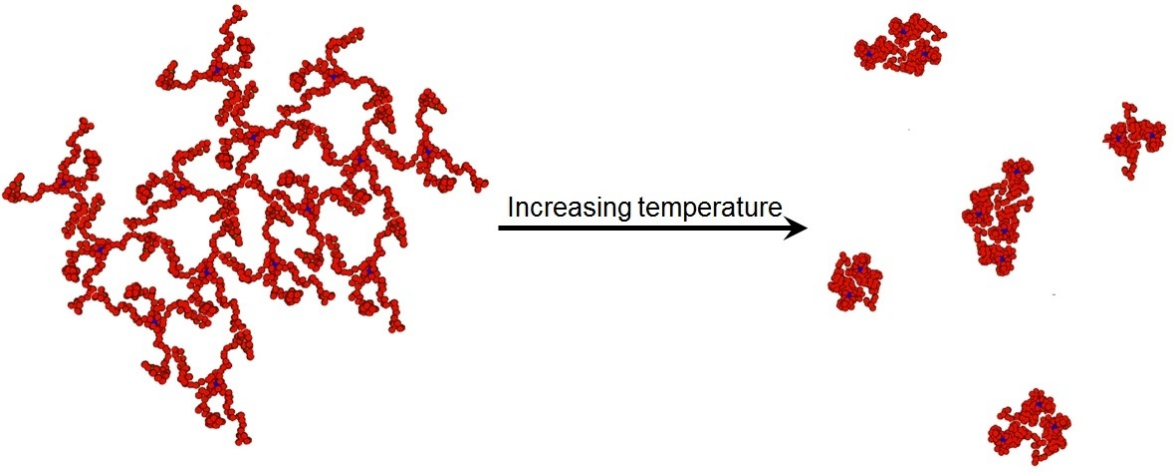
Schematic illustration of the suggested mechanism for fragmentation of the interchain aggregates at high temperatures.

A possible mechanism for the fragmentation at high temperatures is illustrated in Figure 10. At low temperatures, the individual stars have extended conformation, and the aggregates have an open structure. Our conjecture is that as the temperature increases, the solvent conditions become poorer, and the individual star molecules contract. As the single star molecules become smaller, the original open aggregate structure is not able to collapse as a whole unit due to steric hindrance. Our hypothesis is that the structure fragments into several smaller, compact aggregates. After the fragmentation, the entities may start to re‐aggregate into larger clusters again.

## 3 Conclusions

The thermoresponsive PNIPAAM‐star polymers (2‐, 3‐, 4‐, and 6‐arm star polymers) were found to exhibit a complex temperature dependent behavior. Some of the samples form interchain association aggregates even at temperatures significantly below the LCST of PNIPAAM. Aggregation at low temperature is promoted by high concentrations and increasing number of arms. The latter effect is probably related to the corresponding increase in molecular weight when the number of arms is raised. Monte Carlo (MC) simulations illustrate that the individual stars contract as the temperature is raised. Interestingly, the MC simulations also show that this contraction becomes much more pronounced when the number of arms is raised. As the temperature is raised, the solvent conditions become poorer, which causes both contraction and aggregation of the samples. The competition between these two processes leads to a complex size variation as the temperature is increased; aggregation causes the sizes to become larger and contraction simultaneously decreases the sizes. Interestingly, even though the 6‐arm star polymer has the highest tendency to form aggregates at low temperatures; this polymer is less prone to aggregation at temperatures above the cloud point. This is caused by the more compact structure of the 6‐arm star polymer at elevated temperatures, which decreases the sticking probability of the sample.

Several of the samples exhibit a minimum in the turbidity data after the initial increase at the cloud point. The combination of rheology and rheo‐SALS measurements illustrates that this minimum in turbidity data is due to fragmentation of the aggregates (which decreases the turbidity), followed by re‐aggregation (increasing turbidity). High shear rates are found to disrupt the aggregates, especially at low concentrations. The aggregates formed at the highest concentration are more resistant to mechanical forces.

Table 1 summarizes the effect of the number of arms and the concentration on the temperature dependent behavior of the PNIPAAM star polymers. Since the star polymers exhibit a complex associative behavior, the overall tendencies are simplified in the table.

## Conflict of interest

The authors declare no conflict of interest.
